# Barriers and Facilitators to the Implementation of an Electronic Patient-Reported Outcome System at Cancer Hospitals in Japan

**DOI:** 10.7759/cureus.58611

**Published:** 2024-04-19

**Authors:** Yu Uneno, Keita Fukuyama, Ayumi Nishimura, Kana Eguchi, Hideki Kojima, Takeshi Umino, Kikuko Miyazaki, Eiju Negora, Keiko Minashi, Osamu Sugiyama, Taichi Shimazu, Manabu Muto, Shigemi Matsumoto

**Affiliations:** 1 Department of Medical Oncology, Graduate School of Medicine, Kyoto University, Kyoto, JPN; 2 Division of Medical Information Technology and Administration Planning, Kyoto University Hospital, Kyoto, JPN; 3 Department of Health Informatics, Graduate School of Medicine, School of Public Health, Kyoto University, Kyoto, JPN; 4 Department of Real World Data R&D, Graduate School of Medicine, Kyoto University, Kyoto, JPN; 5 Healthcare Solution Department, Marketing Insight Division, INTAGE Healthcare Inc., Tokyo, JPN; 6 Clinical Research Department, Medical Evidence Division, INTAGE Healthcare Inc., Tokyo, JPN; 7 Department of Hematology and Oncology, Faculty of Medical Sciences, University of Fukui, Fukui, JPN; 8 Clinical Trial Promotion Department, Chiba Cancer Center, Chiba, JPN; 9 Department of Informatics, Kindai University, Higashiosaka, JPN; 10 Division of Behavioral Sciences, National Cancer Center Institute for Cancer Control, National Cancer Center, Tokyo, JPN

**Keywords:** implementation facilitators, implementation barriers, treatment, health, patient-reported outcome, cancer

## Abstract

Background and objective

Implementing electronic patient-reported outcomes (ePROs) in oncology practice has shown substantial clinical benefits. However, it can be challenging in routine practice, warranting strategies to adapt to different clinical contexts. In light of this, this study aimed to describe the implementation process of the ePRO system and elucidate the provider-level implementation barriers and facilitators to a novel ePRO system at cancer hospitals in Japan.

Methods

We implemented an ePRO system linked to electronic medical records at three cancer hospitals. Fifteen patients with solid cancers at the outpatient oncology unit were asked to regularly complete the Patient-Reported Outcomes version of the Common Terminology Criteria for Adverse Events (PRO-CTCAE™) questionnaire and European Organization for Research and Treatment Core Quality of Life questionnaire (EORTC QLQ C30) by using the smartphone app between October 2021 and June 2022. Thirteen healthcare professionals were interviewed to identify implementation barriers and facilitators to the ePRO system by using the Consolidated Framework for Implementation Research framework.

Results

The healthcare professionals identified a lack of clinical resources and a culture and system that emphasizes treatment over care as the main barriers; however, the accumulation of successful cases, the leadership of managers, and the growing needs of patients can serve as facilitators to the implementation.

Conclusions

Our experience implementing an ePRO system in a few Japanese oncology practices revealed comprehensive barriers and facilitators. Further efforts are warranted to develop more successful implementation strategies.

## Introduction

Symptom monitoring plays a vital role in oncology practice, as cancer-related symptoms primarily stem from both cancer itself and the adverse effects of anti-cancer treatments [1-4]. Previous studies have shown that assessments by healthcare professionals (HCPs) tend to underestimate these symptoms by 30%-50%, highlighting the need to incorporate patient-reported outcomes into routine clinical practice [5-8].

With the advancements in information technology (IT), the implementation of electronic patient-reported outcomes (ePROs) has become a hot topic of debate [8,9]. Previous research has demonstrated that the implementation of an ePRO system can improve patient health-related outcomes, by prolonging overall survival, improving quality of life (QOL), strengthening patient-HCP relationships, and reducing emergency room visits, hospitalizations, and ICU admissions [10-13]. However, numerous multi-level barriers can hinder the successful implementation of ePRO systems. Recent research has revealed cross-setting universal barriers, such as lack of public subsidies, costs of implementation and sustainment, busy clinics, and lack of IT literacy among patients [8,14].

Moreover, local and culture-specific barriers, such as organizational tension for change, culture of daily symptom management, and underlying organizational structure, can sometimes be critical factors for implementation [15], indicating the need to identify a comprehensive range of barriers and facilitators, including local contextual factors, in the development of implementation strategies that can adapt more effectively to different clinical contexts [15,16]. However, the existing literature on this topic remains limited. Hence, this study aimed to describe the implementation process of the ePRO system and elucidate the barriers and facilitators to the implementation of a novel ePRO system at cancer hospitals in Japan. We believe our findings will contribute to the development of a more effective strategy.

## Materials and methods

This was a qualitative evaluation of barriers and facilitators to the implementation of the ePRO system in oncology practice conducted from October 2021 to June 2022; it involved cancer patients receiving outpatient chemotherapy at Kyoto University Hospital, the University of Fukui Hospital, and Chiba Cancer Center.

Ethical approval

This study was reviewed and approved by the Ethics Committee of the Kyoto University Graduate School and Faculty of Medicine, Kyoto University Hospital (Approval Number: R2965). The study registration number is UMIN000046051. Patients were enrolled in this study after they were fully informed about the study and provided written informed consent.

Patient eligibility criteria

The inclusion criteria were as follows: (1) patients aged ≥18 years receiving outpatient anti-cancer pharmacotherapy (including cytotoxic and molecular targeted agents and immune checkpoint inhibitors) that targeted solid cancers (e.g., gastrointestinal cancer, lung cancer, breast cancer, gynecologic tumor, urological tumor, malignant melanoma, cancer of unknown primary, and orphan cancer) and (2) patients who owned a smartphone (iPhone, Android) and used it daily. The exclusion criteria were as follows: (1) patients with cognitive impairment, (2) patients who could not speak Japanese, and (3) patients considered unsuitable for participation in this study by the researcher (e.g., patients with severe anxiety or depression).

Electronic patient-reported outcome (ePRO) system overview

**Figure 1:**
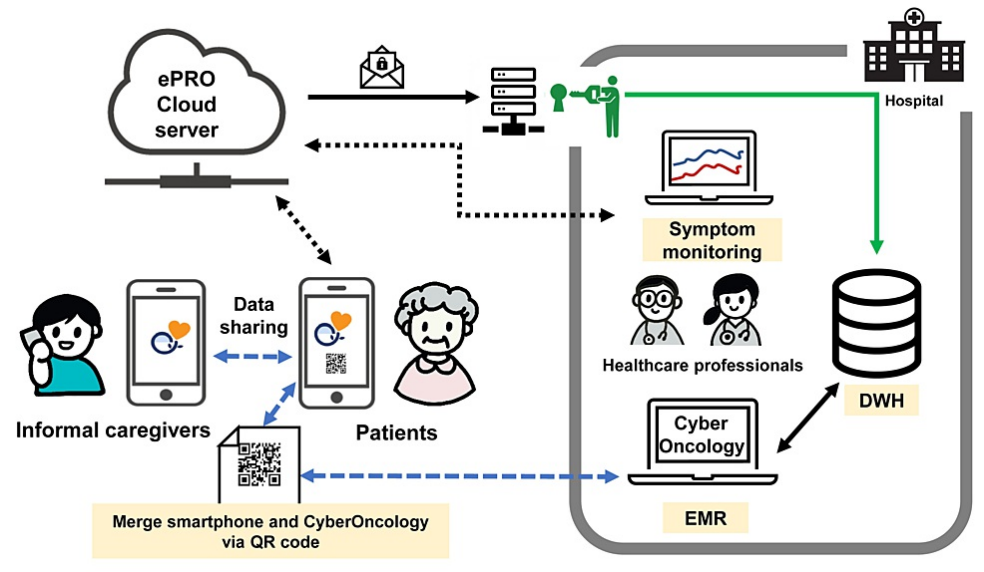
Overview of the electronic patient-reported outcome (ePRO) system for oncology practice An individualized QR code was generated from CyberOncology, which was linked to the electronic medical record, and the patient’s ePRO data and private information were merged. Moreover, the QR code can be generated from a patient’s smartphone and shared with informal caregivers. Anonymized patient data were stored in a cloud server, which could be viewed from within the hospital by connecting to the cloud server via the Internet. In addition, for research purposes, data were accumulated in a data warehouse (DWH) at the study site. Appropriate security was ensured for each system Image credits: Shigemi Matsumoto and INTAGE Healthcare Inc. EMR: electronic medical record; DWH: data warehouse

We have developed a CyberOncology® (CO) tool, which is a structured clinical input support tool linked to electronic medical records (EMRs) for oncology practice [17]. The CO was accompanied by a clinical information template in the EMR, and the collected data were merged on a server within the hospital as linkable, anonymized individual patient data. A CO project was launched in September 2021, to implement and disseminate the system nationwide. In this study, we developed an ePRO system and an eReQo smartphone app, to work in conjunction with our existing CO tool (Figure 1). The ePRO system included patient inputs from their smartphones to the cloud server and in-hospital connections to the server (Figure 1). The ePRO cloud server was not designed to be directly linked to the EMR in hospitals. For activation, an individualized QR code originating from the CO and the EMR can be scanned by using the patient’s smartphone. Informal caregivers, including patients’ family members, can browse the records after recognition by the patients’ smartphone-originated QR code. The patients were followed up for 12 weeks after the enrollment. HCPs can view data in real-time during daily clinical practice (Figure 1).

The eReQo app was equipped with the Japanese version of the Patient-Reported Outcomes version of the Common Terminology Criteria for Adverse Events (PRO-CTCAE) and the European Organization for Research and Treatment Core Quality of Life questionnaire (EORTC QLQ-C30) [18-21]. Patients were requested to answer questions about their condition based on PRO-CTCAE and EORTC QLQ C30 items 29 and 30 (regarding general condition) at least once a week and EORTC QLQ C30 questions 1-28 at least once a month. If a patient did not respond, a daily reminder was issued within the application. The PRO-CTCAE was considered asymptomatic when no symptoms were entered.

In line with the Expert Recommendations for Implementing Change project, we employed 13 pre-implementation strategies [16] (Table 1). In developing the implementation strategies used in this study, the implementation team, researchers, and HCPs discussed the barriers to implementing the ePRO system and examined previous studies on the promotion of ePRO system uptake (Table 1) [8,14].

**Table 1:** Employed implementation strategies We conducted the specific 13 pre- and two post-implementation strategies in line with the Expert Recommendations for Implementing Change (ERIC) project framework [16] ePRO: electronic patient-reported outcome; ERIC: Expert Recommendations for Implementing Change; HCPs: healthcare professionals

Pre-implementation		
Implementation strategy	ERIC domain	Details
1. Investigation of usability and feasibility of prototype smartphone ePRO applications	Conduct cyclical small tests of change	From July 2019, we conducted small-scale tests to investigate the usability and feasibility of prototype smartphone applications
2. Establishment of an industry-academia joint project team responsible for the implementation of the ePRO system	Fund and contract for the clinical innovation	Nippon Telegraph and Denka Co., Ltd. (NTT) worked together to build a platform that enables the use of real-world data, including ePRO, in clinical and research applications. In April 2020, an industry-academia joint course (Department of Real World Data Research and Development) was established at Kyoto University. In addition, to link ePRO with the system for collecting real-world data of electronic medical records by CyberOncology, INTAGE Healthcare Co., Ltd. and Kyoto University entered into a joint research agreement
3. Development of the ePRO system	Develop a formal implementation blueprint	From April 2020 onward, we started designing and developing the ePRO smartphone application and the comprehensive system
4. Participation of data scientists and incorporation of a collaborative research membership related to implementation science	Use data experts. Use data warehousing techniques. Develop academic partnerships	From April 2020 onward, at the beginning of the project, not only clinicians but also data scientists participated in the Kyoto University and INTAGE Healthcare Co., Ltd. project and established a system for the clinical implementation of the ePRO system. In addition, to scientifically plan, practice, and evaluate the implementation of eReQo, we have established a collaborative research team with implementation science experts, qualitative researchers, clinical teams, data experts, and companies
5. On-site needs hearing	Conduct local needs assessment. Capture and share local knowledge	In October 2020, to assess the needs of the implementation site, we interviewed five nurses working in the outpatient oncology unit about the current status and needs for symptom management. The interview disclosed that it was difficult to systematically obtain information on the symptoms of patients, which can lead to the underestimation of patients' symptoms. There was also an opinion that a system that can monitor patients' condition outside the hospital in real-time, not only while in the hospital, is desirable
6. Development of HCP tools	Develop and implement tools for quality monitoring. Develop and organize quality monitoring systems	From January 2021 onward, we started developing symptom-monitoring tools for HCPs and administrators. All operations and input data were recorded as system logs and were designed to enable quality control at any time
7. Participation of HCPs in eReQo development	Inform local opinion leaders. Build a coalition. Promote network weaving	From the eReQo development stage in April 2021, we distributed the eReQo application trial version and provided an opportunity for HCPs to express their opinions to the developers. There was also an announcement that ePRO would be implemented soon and encouraged each HCP to gain a sense of self-efficacy in participating in the project by being involved in its development phase
8. Stakeholder participation	Recruit, designate, and train for leadership	Since December 2020, we have frequently explained and shared the project with the head nurse who manages and supervises the site of the outpatient oncology unit and prepared a system to share the significance of the project and obtain cooperation
9. Manual creation	Develop educational materials. Distribute educational materials	We created a manual for eReQo and administrator tools for patients, families, and HCPs. Those were not distributed in booklets but instead made available electronically on eReQo or from EMR
10. Briefing session for nurses and physicians	Create new clinical teams. Revise professional roles. Develop resource-sharing agreements	From July 2021, we have held multiple briefing sessions on the project for nurses who are in charge of patient care. There was an opinion among HCPs that the actual feeling and texture of use were unclear just by explaining the outline of the project; thus, we decided to prepare a demonstration machine in the outpatient oncology unit before the start of implementation. In October 2021, we held a briefing session on the project for physicians. At that time, we presented the procedure to be performed by the physicians in the form of a checklist and explained the available materials for patient registration. At that time, there were requests, such as the creation of a quick manual for patients
11. Preparing the eReQO demo machine	Make training dynamic. Conduct ongoing training	We prepared five smartphones equipped with the eReQo application and prepared them so that nurses and physicians could introduce them to their patients appropriately in the outpatient oncology unit. When necessary, briefing sessions were held for nurses to explain the outline of the project and how to operate it
12. Build an interactive assistance system	Centralize technical assistance. Facilitate technical assistance. Provide clinical supervision. Provide ongoing consultation	Even after the implementation of the system, if any problem or question arose, we informed the point of contact for HCPs to seek assistance via e-mail, telephone, or verbally
13. Preparation for expansion to other facilities	Stage implementation scale-up	From the middle of 2021 onward, we introduced eReQo to other hospitals to participate in the study. From October 2021, briefing sessions have been held as needed for other hospitals that wish to participate
Post-implementation		
Implementation strategy	ERIC domain	Details
14. Employment of patient enrollment assistants	Remind clinicians	After kick-off in October 2021, patient enrollment has been poor due to the large number of concurrent clinical studies and busy oncology clinics. Therefore, we hired patient enrollment assistants and asked them to take charge of picking up candidate patients, preparing documents related to enrollment, and making announcements to physicians
15. Sharing successful cases using the ePRO system	Remind clinicians. Purposely reexamine the implementation	For further refinement of reminders and interventions for clinicians, we introduced successful cases of using eReQofor physicians

Qualitative evaluation of the practical context of the ePRO system implementation

Semi-structured interviews were conducted with HCPs involved in the implementation of the ePRO system to systematically identify the barriers and facilitators to implementing the ePRO system. The interviews were conducted by the principal investigator, and the audio-recorded interviews were transcribed. A deductive content analysis was performed according to the Consolidated Framework for Implementation Research (CFIR) by two independent researchers (Y.U. and M.N.) [15,22]. CFIR is a framework used in many implementation studies, comprising five domains and 39 items: “intervention characteristics,” “external setting,” “internal setting,” “individual characteristics,” and “process.” The CFIR covers the perspectives for identifying factors of barriers and facilitators to implementation [15,22]. To ensure rigor and trustworthiness, experienced investigators (K.M. and T.S.) supervised and examined the validity and consistency of the results. To further strengthen the credibility of the results, a detailed preliminary table with a summary figure was shown to all interviewees to confirm that the views of HCPs were appropriately reflected.

Evaluation of the ePRO system usage logs

To clarify adherence, we used the patient ePRO system usage log data (including PRO-CTCAE and QLQ-C30 data), as these were extractable and informative data within our ePRO system [23].

Statistical analyses

Descriptive statistics were performed on the background information of the patients and HCPs who were interviewed. Patient input logs were converted into simple longitudinal plotting data. To classify the patient behavior regarding eReQo usage, we calculated the following mean value per week for both the input and reminder. The input was the total number of inputs for CTCAE and QOL divided by the participating period. The reminder was the total number of reminders for the PRO-CTCAE and QOL divided by the participating period. The classification cutoff values were defined post hoc based on a visual assessment of the distribution of the plots of the eReQo application usage logs.

The analysis was conducted using R [24] (version 4.1.2)/RStudio [25] (version 2023.6.2.561) on Windows Subsystem Linux/Windows 10 Home edition. The codes used in our study were reposited on GitHub (https://github.com/fk506cni/ereqo_log_calc). The main package used was Tidyverse [26]. Data.table [27] was used for data handling. Supplementary Table1 [28] and openxlsx [29] were used for table output. Ggplot2 [30], ggpubr [31], ggh4x [32], ggnewscale [33], and officer [34] were used for visualization.

## Results

After the implementation, the increase in ePRO usage was slow, and two implementation strategies were added (employment of patient enrollment assistants and sharing of successful cases using the ePRO system) (Table 1).

Patient characteristics

The patient characteristics are presented in Table 2. A total of 15 patients were enrolled at three hospitals. The Eastern Cooperative Oncology Group Performance Scale (ECOG PS) score was 0 for the majority of the patients (80.0%), and the mean age of the patients was 61.2 years. Pancreatic cancer was the most common type of cancer (26.7%).

**Table 2:** Patient characteristics SD: standard deviation; ECOG PS: Eastern Cooperative Oncology Group Performance Status

	Overall	Heavy_user	Light_user	Low_responder
	(N=15)	(N=5)	(N=4)	(N=6)
Age, years				
Mean (SD)	61.2 (10.5)	66.7 (11.8)	63.7 (8.60)	55.0 (8.55)
Median (min, max)	58.4 (48.4, 78.3)	72.8 (51.6, 78.3)	64.6 (53.6, 71.9)	52.1 (48.4, 70.5)
Sex, n (%)				
Female	6 (40.0%)	2 (40.0%)	1 (25.0%)	3 (50.0%)
Male	9 (60.0%)	3 (60.0%)	3 (75.0%)	3 (50.0%)
Primary cancer site, n (%)				
Pancreas	4 (26.7%)	1 (20.0%)	0 (0%)	3 (50.0%)
Stomach	3 (20.0%)	2 (40.0%)	0 (0%)	1 (16.7%)
Duodenum	1 (6.7%)	1 (20.0%)	0 (0%)	0 (0%)
Cervix	1 (6.7%)	0 (0%)	1 (25.0%)	0 (0%)
Biliary tract	1 (6.7%)	0 (0%)	1 (25.0%)	0 (0%)
Soft tissue	1 (6.7%)	0 (0%)	1 (25.0%)	0 (0%)
Derma	1 (6.7%)	0 (0%)	1 (25.0%)	0 (0%)
Colorectum	1 (6.7%)	0 (0%)	0 (0%)	1 (16.7%)
Head and neck	1 (6.7%)	0 (0%)	0 (0%)	1 (16.7%)
Others	1 (6.7%)	1 (20.0%)	0 (0%)	0 (0%)
Institution, n (%)				
Kyoto University Hospital	11 (73.3%)	3 (60.0%)	3 (75.0%)	5 (83.3%)
University of Fukui Hospital	3 (20.0%)	1 (20.0%)	1 (25.0%)	1 (16.7%)
Chiba Cancer Center	1 (6.7%)	1 (20.0%)	0 (0%)	0 (0%)
ECOG PS, n (%)				
0	12 (80.0%)	4 (80.0%)	4 (100%)	4 (66.7%)
1	3 (20.0%)	1 (20.0%)	0 (0%)	2 (33.3%)

Qualitative evaluation of the practical context of the ePRO system implementation

**Figure 2:**
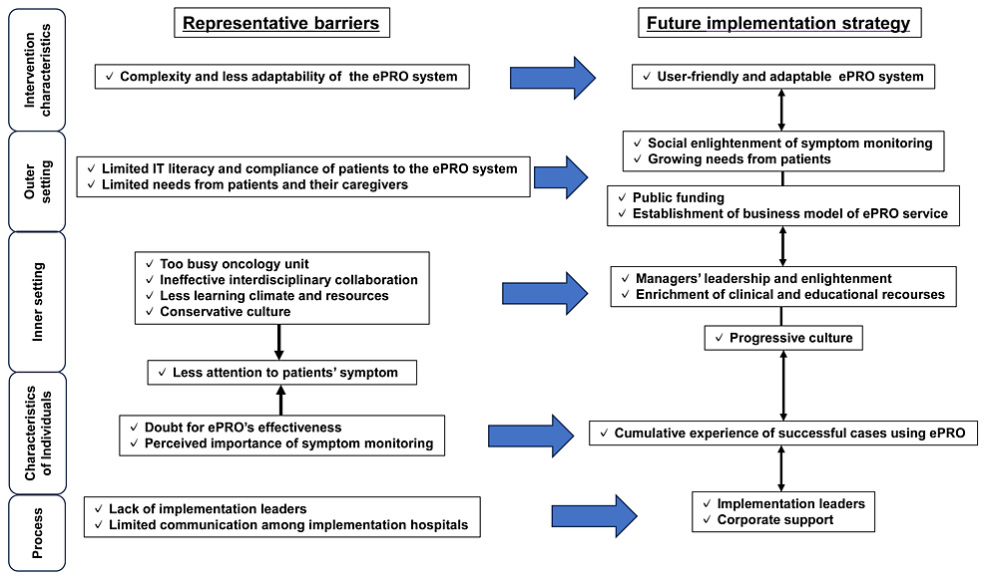
Summary of barriers and future strategies related to the ePRO system implementation in Japanese clinical context ePRO: electronic patient-reported outcomes

We interviewed 13 HCPs involved in the implementation of the ePRO system at three hospitals; six (46.2%) were women, six (46.2%) were nurses, six (46.2%) had board certifications, and five (38.4%) were managers (Table 3). The characteristics of the interviewees are shown in Table 3, and a summary of the interviewees’ consensus is presented in Figure 2.

**Table 3:** Characteristics of the interviewees SD: standard deviation

Variables	Overall
	(N=13)
Age, years	
Mean (SD)	46.2(7.6)
Median (min, max)	43 (33, 60)
Sex, n (%)	
Female	6 (46.2%)
Male	7 (53.8%)
Occupation, n (%)	
Physician	7 (53.8%)
Nurse	6 (46.2%)
Board certifications, n (%)	
Yes	6 (46.2%)
No	7 (53.8%)
Management role, n (%)	
Yes	5 (38.4%)
No	8 (61.5%)

Intervention characteristics

Intervention characteristics are detailed in Table 4. Several HCPs supported the use of the ePRO system. For example, they highlighted improvements in various outcomes, including enhanced communication, more efficient workflow, reduced patient emergency visits, and the hope for a user-friendly ePRO system. Specific features such as the automatic transfer of symptom records to the EMR, the possibility of modifying query items, the import of clinical images, and the reduction in labor required for patient searches were identified as useful features for inclusion. In addition, concerns were expressed that the introduction of new technology would place a burden on new sites and cause other problems (e.g., troubleshooting with ePRO systems and lawsuits due to unconfirmed ePRO data).

**Table 4:** Barriers and facilitators to the implementation of an ePRO system for oncology practice: “intervention characteristics” ePRO: electronic patient-reported outcome; HCPs: healthcare professionals

Domain	Barriers - summary	Facilitators - summary
B. Evidence strength and quality		There is an accumulation of scientific evidence on the benefits of implementing an ePRO system
C. Relative advantage	Inability to make handwritten notes, such as self-care notes, with the ePRO application. Symptoms are not identifiable using ePRO (some symptoms are identifiable only with face-to-face consultation). There are no specific problems in the operation of the self-care note, and there are no inconveniences. Focusing on the ePRO system only, it is difficult to communicate with the patient while making eye contact. Compared with a self-care note, there is an increased burden of explaining, troubleshooting, and responding to ePRO systems. In the ePRO system, responsibility for loss, failure, and power supply of smartphones can occur. In the ePRO system, there can be an increase in the risk of troubles with patients and lawsuits due to unconfirmed ePRO data	The ePRO system can identify symptoms that tend to be overlooked with self-care notes or that are of interest to patients. With the ePRO system, it is possible to reduce the workload of HCPs, such as symptom recording on EMR, loss of self-care notes, and explanation of self-care note usage. Using the ePRO system increased communication between patients and HCPs regarding their symptoms. Using the ePRO application, clinical benefits can be expected, such as a reduction in emergency visits. Compared with ePRO, self-care notes may have poorer visibility, such as messy characters. The ePRO system makes it easier to confirm long-term symptom trends. There is a high cost of creating self-care notes
D. Adaptability	There is an inability to record image data. With ePRO, it was difficult to change questions and survey items. There is an inability to alert for symptoms above the threshold	Simultaneous operation of the self-care note and the ePRO system can make it easier for patients to select tools that are easy to use. Informal caregivers can help patients with ePRO input. Hospital staff support for the use of the ePRO application
F. Complexity	The ePRO system application is cumbersome and complex. Patients cannot operate ePRO when they want to record ePRO, such as when symptoms are too severe. With a self-care note, just opening it is enough, but with the ePRO system, patients needed help with loading the PC and searching. The concurrent operation of self-care notes and the ePRO system complicated the work	
G. Design quality and packaging	The ePRO system did not show a proposal of specific coping actions. There was no specification to transfer symptoms to electronic medical records. It was difficult to use the ePRO application from the patient's point of view, such as too many items and inappropriate timing of reminders, and the patient did not feel attached to the application. The medical staff had difficulties using the monitoring tools, such as the ePRO system not being linked to electronic medical records	Even people who cannot tell their symptoms simply can understand what happened in real-time at a glance with the ePRO application. There is improvement in the usability of monitoring tools for healthcare providers

Outer settings

Outer settings are detailed in Table 5. HCPs pointed out that patient needs, compliance, and IT literacy might be limited. Providing patient information and education and providing patients with the opportunity to discover benefits through hands-on experience were cited as facilitators to increase their needs. In addition, information sharing among cancer hospitals, cooperation with companies, monetization, public subsidies, and legal regulations affected the ePRO system implementation.

**Table 5:** Barriers and facilitators to the implementation of an ePRO system for oncology practice: “outer setting” ePRO: electronic patient-reported outcome; HCPs: healthcare professionals; IT: information technology

Domain	Barriers - summary	Facilitators - summary
Ⅱ. Outer setting		
A. Patient needs and resources	Poor patient compliance with ePRO use. Patient's limited IT literacy. Limited opportunities to inform and educate patients about ePRO. Patient's preference to communicate with HCPs face-to-face, rather than electronically. Failure in causing patients to find benefits in using ePRO and increase needs	High IT literacy of patients. Support and requests from informal caregivers for the use of ePRO
B. Cosmopolitanism		Opportunity to share information on the status of symptom monitoring with external cancer hospitals
C. Peer pressure		Increasing momentum for ePRO implementation among HCPs and companies
D. External policy and incentives	Un-establishment of the business model regarding ePRO. Lack of public subsidies, such as medical fees from the national healthcare insurance. Legal regulations, such as the Personal Information Protection Law and policies of each institution regarding privacy	

Inner settings

Inner settings are described in Table 6. HCPs highlighted the fact that they have limited opportunities to acquire skills related to symptom management and that even if they do, they are in an environment and culture that makes it difficult to demonstrate them: *A board-certified nurse (ID 4-18): I sometimes stop delivering specialized care by myself, even though no one has stopped me (…) It is because nurses work in teams, so there is a culture of tacit understanding. Therefore, it is difficult to put into practice the various knowledge obtained at schools and academic conferences. Ultimately, this may reduce HCPs' interest in symptom management.* Furthermore, it was noted that the prevailing culture and environment prioritize the delivery of treatment, such as administering intravenous infusions of anti-cancer drugs over identifying unexpressed patients’ symptoms. Furthermore, the enrichment of clinical resources and the leader's initiative for symptom management were identified as factors promoting multidisciplinary care delivery.

**Table 6:** Barriers and facilitators to the implementation of an ePRO system for oncology practice: “inner setting” ePRO: electronic patient-reported outcome; ERIC: Expert Recommendations for Implementing Change; HCPs: healthcare professionals

Domain	Barriers - summary	Facilitators - summary
A. Structural characteristics	The work shift system of nurses makes it difficult to monitor the patient's condition. A personnel system in which nurses who have acquired skills are regularly transferred to other departments	
B. Networks and communications	Failure to share and discuss patient symptoms, conditions, and treatment plans among HCPs	System for information sharing regarding patient symptoms, conditions, and treatment plans among HCPs
C. Culture	The culture emphasizes medical management rather than care and nursing. The culture of the sticking out is stakes-driven, which makes it difficult for individuals to demonstrate specialized skills. Culture of resistance to the introduction of new technologies	Culture of HCPs doing their best to provide patient care
D. Implementation climate		
2. Compatibility		Leaders' positive attitude toward the introduction of new technologies and enhancing symptom monitoring
3. Relative priority	Work pressures to prioritize medical management over care and nursing. Attitudes to prioritize other tasks over symptom monitoring	
6. Learning climate	Leaders' reluctant attitude to adopt and learn new things. Leaders' unwillingness to change, learn, and introduce new technologies	Building a system for sharing each HCP's clinical practice and a system for consultation when problems arise
E Readiness for implementation		
1 Leadership engagement	Lack of awareness of the importance of symptom monitoring	
2 Available resources	Insufficient human resources to support ePRO operation. Busy clinical practice. Reduced time for education/training or inability to secure opportunities	Securing opportunities for education and training inside and outside the hospital
3 Access to knowledge and information		Access to ePRO user manuals and opportunities to use demo machines

Characteristics of individuals

Characteristics of individuals are presented in Table 7. HCPs reported that their perception of the importance of symptom monitoring and ePRO was dependent on various factors, such as clinical experience, patient needs, individual position, IT literacy, and aptitudes.

**Table 7:** Barriers and facilitators to the implementation of an ePRO system for oncology practice: “characteristics of individuals” ePRO: electronic patient-reported outcome; HCPs: healthcare professionals; IT: information technology

Domain	Barriers - summary	Facilitators - summary
Ⅳ. Characteristics of individuals		
A. Knowledge and beliefs about the intervention	Concerns about the significance of the ePRO system. Limited IT literacy among HCPs	Perceived significance of the ePRO system
C. Individual stage of change	Insufficient clinical experience and unawareness of the importance of symptom monitoring. Learned helplessness to contribute to symptom management	Cumulative experience that HCPs can effectively use the ePRO system in clinical practice. Active use of ePRO by patients. Recognize the importance of symptom monitoring through clinical experience, certification, and knowledge acquisition
E. Other personal attributes	Lack of aspiration of HCPs	Being qualified makes it easier to take initiative in symptom management within the organization

Process

The process is illustrated in Table 8. The project manager reported that the lack of allocation of human resources in the process affected the implementation. Concentrating resources, particularly in the early phase of implementation, was identified as a critical need to gain momentum.

**Table 8:** Barriers and facilitators to the implementation of an ePRO system for oncology practice: “process” ePRO: electronic patient-reported outcome; HCPs: healthcare professionals

Domain	Barriers - summary	Facilitators -summary
A. Planning	Failure to establish a population suitable for ePRO use	Allocation of human resources to support and follow up ePRO operation and symptom management. Clarification of the division of roles between nurses in charge of cancer treatment and in charge of care/nursing
B. Engaging		
1. Opinion leaders		Presence of opinion leaders
2. Formally appointed internal implementation leaders	Failure to secure a leader to lead the implementation	
3. Champions	Failure to secure champions to enhance the implementation	
4. External change agents		The presence of an external company to assist with implementation
C. Executing	Failure to catch up with the early introduction of the ePRO system. The project manager is unable to share progress and communicate face-to-face with multiple facilities (partly due to the coronavirus disease 2019 pandemic)	

ePRO usage log evaluation

**Figure 3:**
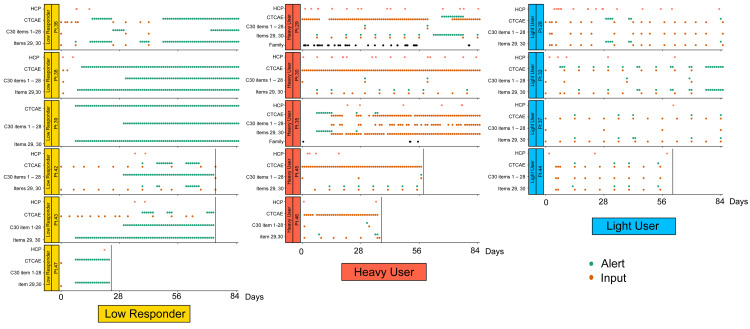
eReQo system usage logs for patients, informal caregivers, and HCPs 15 study participants showed three different behaviors, which are defined as "low responder (n=6, yellow)", "heavy user (n=5, orange)" and "light user (n=4, blue)." Each column represents the individual study participants' behaviors, with the horizontal axis as time course and the vertical axis as HCPs monitoring, CTCAE, and EORTC QLQ 30 responses CTCAE: Patient-Reported Outcomes version of the Common Terminology Criteria for Adverse Events; EORTC QLQ C30: European Organization for Research and Treatment Core Quality of Life questionnaire; HCPs: healthcare professionals

**Figure 4:**
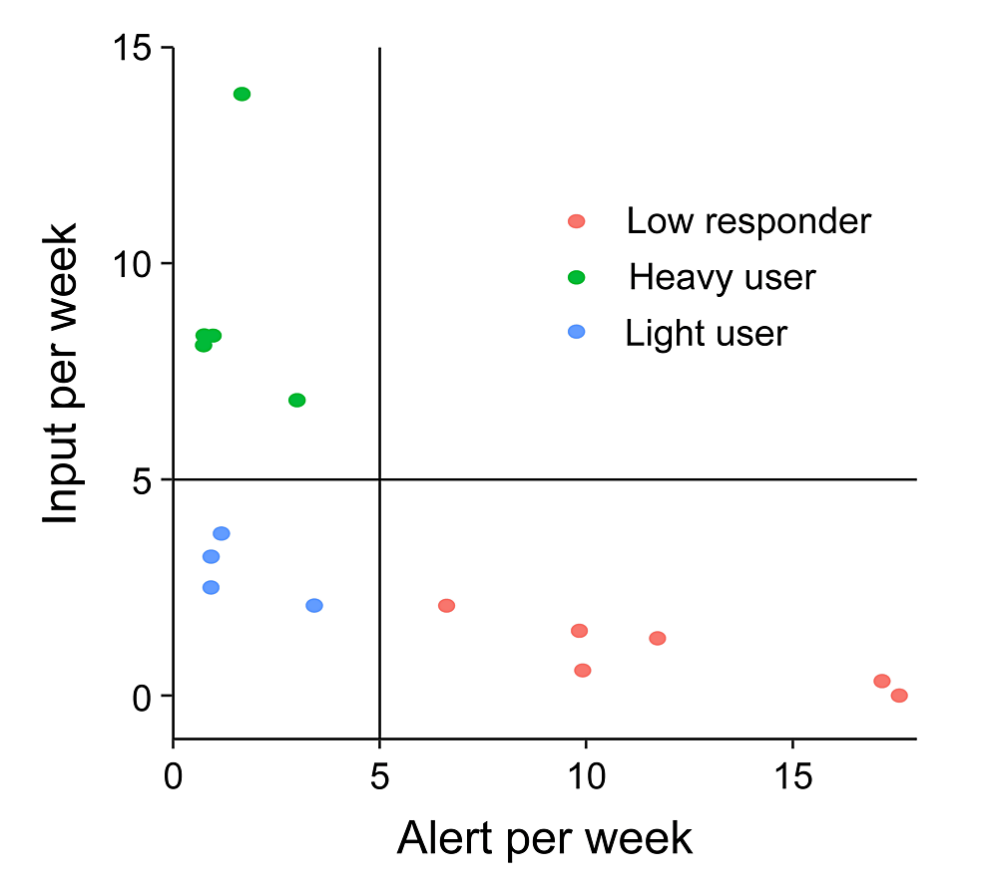
Classification based on eReQo log data Classification cutoff values were defined post hoc based on a visual assessment of the distribution of the plots

The ePRO usage logs of the 15 patients who participated in the study are shown in Figure 3. During the 12-week follow-up period, the participants used reminders in three ways: almost every day, rarely, or in response to reminders. Interestingly, the frequency at which clinicians reviewed ePRO data also varied significantly. The visualization of the distribution of inputs and reminders revealed a split distribution of patient behavior, with a cutoff of more than or less than five times per week. For this reason, we defined the following three groups: heavy user, light user, and low responder (Figure 4). Regarding the characteristics of the three groups, older users tended to be more prevalent in the heavy user group than in the other two groups (Table 2). Notably, two patients in the heavy user group allowed their family members to use the app (Figure 3, Patients 29 and 35).

## Discussion

This study described the implementation of an ePRO system in Japanese cancer hospitals, revealing the provider-level implementation barriers and facilitators and shedding light not only on the challenges posed by limited clinical resources and budgets but also the unique contextual factors within oncology practice (Figure 2).

Our comprehensive qualitative analysis revealed the need for additional implementation strategies beyond the ones we already adopted (Table 1). While financial support, national health insurance premiums, and hospital managers' mandatory directives could be powerful facilitators for implementation [35,36], modifying these factors may prove challenging and beyond the purview of individual HCPs. Two important issues were highlighted in the interviews: the significant effect of a culture of teamwork and HCP perspectives regarding symptom management on implementation and insufficient development of an effective ePRO system (Tables 2-6). Furthermore, it was suggested that each implementation strategy did not work independently but they were interrelated, resulting in a synergistic effect (Figure 2). Among these strategies, the accumulation of successful small-scale cases emerged as a crucial catalyst within oncology practice, with the potential to synergize all strategies toward normalization [37]. Moreover, revising implementation strategies with more frequent formative evaluations during the implementation phase will lead to further success [38].

In the quantitative analysis of patient adherence, we classified patient ePRO usage into three distinct patterns. Patient adherence to ePRO may be influenced by a multitude of factors, including not only IT literacy but also general physical and emotional conditions, belief in the ePRO system, symptom communication, and individual patient personality (Table 3). From the perspective of future digitalization in medicine, it would be desirable to implement the ePRO system as a default (normalization) setup using behavioral economic techniques, such as nudges [39,40]. However, in some populations, analog operations are necessary and should be allowed as an exception. In our study, we found considerable variability in the viewing status of HCPs. Low utilization of ePRO by HCPs can diminish patient compliance [41-43], emphasizing the need for a comprehensive and mutually synergistic implementation strategy that actively involves HCPs.

This study has some limitations. Firstly, the sample size of 15 participants was relatively small, and the depth of activities during the predefined research period was affected by contractual constraints and the coronavirus disease (COVID-19) pandemic. The spread of COVID-19 has paradoxically created external pressure to implement the ePRO system, and a more formative evaluation of the strategy to accelerate momentum would lead to more successful implementation [37,44]. Secondly, this study was conducted at high-volume centers, such as university hospitals and cancer centers in urban areas, potentially limiting the generalizability to other contexts, such as rural general hospitals. Finally, this study was supported by corporate investment, which may paradoxically limit its dissemination potential. To cover implementation expenses, financial support, such as national health insurance support or the establishment of a business model for the secondary use of clinical informatics, is a critical concern.

## Conclusions

We identified comprehensive barriers and facilitators to the implementation of an ePRO system in Japanese oncology practice. The HCPs identified a lack of clinical resources and a culture and system that emphasizes treatment over care as the main barriers; however, the accumulation of successful cases, the leadership of managers, and the growing needs of patients can be facilitators to the implementation. Further research to develop more successful implementation strategies is warranted.
